# Effect of home-based comprehensive geriatric assessment on hospital days in multimorbid older adults: a randomized controlled trial (the GerMoT trial)

**DOI:** 10.1007/s41999-026-01537-4

**Published:** 2026-07-06

**Authors:** Karol Robert Biegus, Anna Saxne Jöud, Katarina Steen Carlsson, Anne Wissendorff Ekdahl

**Affiliations:** 1 Department of Geriatric Medicine, Helsingborg Hospital, Charlotte Yhléns Gata 10, 251 87 Helsingborg, Sweden; 2 Department of Clinical Sciences, Helsingborg, Faculty of Medicine, Lund University, Lund, Sweden; 3 Department of Clinical Sciences, Lund University, Lund, Sweden; 4 Department of Laboratory Medicine, Occupational and Environmental Medicine, Lund University, Lund, Sweden; 5 Department of Research, Development, Education and Innovation, Skåne University Hospital, Lund, Sweden; 6 The Swedish Institute for Economics, Lund, Sweden; 7 Department of Clinical Sciences, Malmö, Lund University, Lund, Sweden

**Keywords:** Comprehensive geriatric assessment, Mobile, Hospitalisation, Frailty

## Abstract

**Purpose:**

To determine whether Comprehensive Geriatric Assessment (CGA)-based healthcare, delivered by a mobile team over 24 months in patients’ homes reduces hospital care or improves quality of life, physical performance, frailty, activities of daily living (ADL), or cognition and whether it reduces mortality or number of hospital admissions.

**Methods:**

Patients ≥ 75 years of age, with ≥ 3 visits to the Emergency Department in the last 18 months, with ≥ 3 diagnosed diseases, living in their own home and within the catchment area of the local hospital were invited to the study. Enrolled patients were randomized to either the intervention group, which received CGA-based healthcare by a geriatric mobile team in addition to usual healthcare, or a control group which received usual healthcare only.

**Results:**

450 participants were randomised equally into the intervention and the control groups. The participant’s mean age was 83 years (SD = 5.5) and 245 were female (54%). The mean number of days spent in hospital did not differ between the intervention and control groups after 24 months (16.2 and 17.6 days; *P* = 0.14) follow-up. There were also no differences in mortality, quality of life, frailty levels, or other health-related outcome measures.

**Conclusion:**

This study did not show any clinically relevant effect of home CGA-based healthcare delivering over 24 months on the outcomes measured. We question the quality of the intervention and speculate that lack of continuity and scarce team building could have contributed to the results.

**Trial registration:**

The trial was registered at ClinicalTrials.gov in year 2016 with ID: NCT02923843.

## Key summary points

**Aim:**

Does home‑based healthcare using a structured, multidisciplinary Comprehensive Geriatric Assessment (CGA) approach for multimorbid older adults with high healthcare use reduce hospital care and improve broader health outcomes?

**Findings:**

There were no observed effects on hospital care or other health outcomes.

**Message:**

The results suggest that adding home CGA-base healthcare to usual care does not reduce hospital care or improve health outcomes if not combined with proper team building of the healthcare team.

## Introduction

Most healthcare systems are designed to respond to symptoms and single illnesses. The following treatment typically targets one acute condition and works well in those situations. However, as the population ages, an increasing number of people live with multiple chronic conditions [1, 2] resulting in more unplanned care, frequent hospitalisations, and increased strain on healthcare systems [3, 4]. This shift has driven a need to reorganise healthcare processes for people with multiple simultaneous chronic illnesses [5, 6]. Integrating available care and social services into holistic patient care plans is essential. Leveraging patients’ social networks, including family, friends, and community organisations further enhances care. To achieve this and shift from specialty-based single-organ healthcare, geriatric care uses Comprehensive Geriatric Assessment (CGA), a form of healthcare that is more suited to multimorbid older patients. CGA is a holistic, multidimensional, interdisciplinary diagnostic and treatment process used to address the medical, psychological, and functional needs of frail older people. Integral to CGA is an emphasis on planning and proactive interventions [7]. CGA can be performed during an inpatient stay but also as an outpatient intervention or even in patient’s homes through a mobile team. The main barriers to implementing CGA-based healthcare are concerns about increased costs and the need for interprofessional teamwork [8].

Sweden’s healthcare system is decentralized, taxpayer-funded, with universal coverage and has a robust governance [9]. The system is managed by 21 regions, responsible for hospitals and primary care. There are 290 municipalities overseeing all community services, social care, and all primary healthcare delivered in patient’s homes including nursing home health and social care if needed [9]. Sweden has approximately 2 inpatient beds per 1000 inhabitants compared to a European average of 4, 4 beds [10, 11]. This puts high pressure on the available beds and directs focus towards their optimal use.

Older people with multimorbidity often require inpatient hospital care to manage exacerbations of chronic diseases or to diagnose and treat new conditions [12]. However, hospitalisation may serve as a marker of unmet healthcare needs in the outpatient setting. Such a hospitalisation is referred to as an avoidable hospitalisation. The Swedish National Board of Health and Welfare defines hospitalisations as avoidable if they could have been prevented through effective primary care and community-based care, and these include hospitalisation for certain acute and chronic conditions [13]. The rate of avoidable hospitalisations in an area is thought to reflect access to and quality of primary healthcare, social care but also sociodemographic factors and health behaviours [13]. In 2023, the Swedish National Board of Health and Welfare recorded ~ 4400 avoidable hospitalisations per 100,000 hospitalisations in patients aged ≥ 65 years [13]. These should, if possible, be avoided due to the associated risks of hospitalisation including fractures, medication errors, delirium, iatrogenic infections, and further disabilities [14]. Given the complex needs of older people and known holistic approach of CGA, could CGA in the outpatient setting, in addition to usual primary healthcare, reduce avoidable hospitalisations?

In the acute inpatient hospital setting, CGA has been studied in numerous randomised controlled trials (RCT) and systematic reviews [7, 15]. This research shows that patients who received CGA-based healthcare in this setting were less likely to require nursing home admission, more likely to have improved functional and cognitive capacity, and had a lower mortality [7, 15].

In contrast, in the outpatient setting, CGA-based healthcare has not shown the same results as for inpatient care [8, 16]. One review by Briggs et al. showed no impact on mortality or nursing home admission and concluded that further studies are needed to assess the effect on hospital admissions, quality of life, and functional status [16]. The second review by Sum et al. included 43 studies of outpatient CGA, including 18 studies of CGA delivered in patient’s homes. Only one of these home base interventions included a geriatrician. Their overall conclusion was that of mixed evidence on the health outcomes of CGA on community-dwelling older adults.

The one trial that has specifically assessed home-based CGA that included a geriatrician, AGe-FIT [17], showed superior results of home CGA-based healthcare compared to usual care with respect to days in hospital, sense of security, and mortality [18]. In the present trial, we aimed to test if CGA-based home healthcare can be equality effective in a different region of Sweden.

We evaluated the effects of CGA-based home healthcare, delivered by a mobile team over 24 months, on the number of days spent in hospital. Secondary outcomes included mortality, quality of life, number of hospital admissions, physical performance, frailty, activities of daily living (ADL), and cognition.

## Methods

### Trial design

The GerMoT trial was a single-centre, randomized controlled trial with baseline assessor blinding. The trial was registered at ClinicalTrials.gov in year 2016 with ID: NCT02923843. Participants were randomized 1:1 to either the intervention group (IG), receiving home CGA-based healthcare in addition to usual healthcare, or the control group (CG), receiving only usual healthcare.

### Changes to trial design

During the recruitment process, the inclusion criteria were expanded from 12 to 18 months for Emergency Department visits. This was done to increase the number of patients recruited as we struggled to meet the number of participants needed as per the power calculation.

### Trial setting

The trial was conducted from the Geriatric Medicine Department of Helsingborg Hospital; a medium-sized hospital with 300 inpatient beds in southern Sweden, serving approximately 215 000 inhabitants in both urban and rural areas. The hospital had 24-h emergency admissions for surgical and medical emergencies. The Geriatric Mobile Team operated from the recently opened Geriatric Department and there were no other geriatric outpatient services in the area.

### Participants and recruitment

Following ethical approval, the recruitment process started. Lists of eligible individuals were obtained from the Patient Administrative Register of Care in Region Skane [19]. Patients meeting the following inclusion criteria were included in the study: ≥ 75 years of age, ≥ 3 visits to the Emergency Department in the last 18 months, ≥ 3 diseases diagnosed according to different ICD-10 (International Statistical Classification of Diseases and Related Health Problems, 10th revision) chapters, living in their own home and within the catchment area of Helsingborg Hospital. Participants lived in their own home with or without social care.

The recruitment process has been described in detail elsewhere [20, 21]. In short, each eligible individual received a letter with information about the study and approximately 1 week later was contacted via phone by a study nurse. Upon preliminary consent, a home visit was planned for obtaining written informed consent and collecting data.

### Sample size

A power calculation was performed based on the primary outcome—number of inpatient days over 24 months. Based on a similar trial [18], the expected difference between the intervention and control groups to be 4.1 days with a standard deviation of 15 days in both groups. To detect a significant difference between the groups with a two-tailed test and the significance level at 0.05 and 80% power, we needed 211 participants in each group. A total of 450 participants were recruited, with 225 in the intervention group and 225 in the control group.

### Randomisation

Simple randomisation was performed. No restrictions were applied. A master list with 450 numbers was created by the Principal Investigator (*AE*). After baseline measurement, the participants were randomized 1:1 to the intervention or control group using a computer package (IBM SPSS Statistics version 23.0).

Only the intervention group participants were contacted by a GerMoT nurse for further procedures as described in the intervention. The master list was kept by a trial administrator who was not involved in the recruitment of participants.

### Blinding

Each individual’s datum was initially identified and extracted from the administrative data base PASIS by blinded administrative personnel not involved in the trial. All pre-randomisation baseline data were collected by blinded assessors/interviewers, who did not participate in the patient’s healthcare before or after randomisation. Participants were informed of their assignment to the intervention group or the control group after all baseline data had been collected but prior to first contact with the mobile team.

### Intervention

#### Intervention group (IG)

The intervention group received, in addition to the same usual healthcare as the control group, healthcare provided according to the principles of CGA by a team consisting of a physician, a nurse, a physiotherapist, an occupational therapist, and a pharmacist. The intervention included:

1. A nurse visited participants at home and performed a structured interview that had a holistic character and included questions about the patient’s life and health (see supplement 1). In addition, a venous blood sample was drawn for analysis of haemoglobin, white blood cell and platelet count, pro-B-type natriuretic peptide, blood glucose, creatinine, sodium, potassium, and c-reactive protein.
2. A structured medication review was performed by a clinical pharmacist who phoned the patient and if necessary, their relatives. The pharmacist had access to the patient’s medical records and a Prescribed Drug Register. The pharmacist compiled a current medication list, provided recommendations regarding doses, interactions, possible side effects, and potentially inappropriate medications, and noted the patient’s wishes. The pharmacist’s recommendations were recorded in the patient’s medical records.
3. The patient was invited for a first consultation with the physician at the hospital. Prior to the visit, the physician reviewed the medical records. The patient was encouraged to bring a next of kin to the visit. During the appointment, the physician confirmed the medical history and current health problems, and blood results were discussed. The physician then performed a physical examination, including neurological assessment and an electrocardiogram was recorded. The visits concluded with the physician summarising all assessments, adjusting the current medication list and planning follow-up or further investigations as needed. The patient was handed a printout of their current medication list (or one was sent by mail within a few days).
4. Each patient was discussed at an interdisciplinary meeting. Such meetings were held by the entire team once or twice a week to discuss new patients recruited to the trial. The team summarised the patient’s physical, psychological, social, and functional situation, focusing on actions to improve quality of life. Further actions were planned, if necessary, such as an assessment by a dietician, home visits by the occupational therapist or the physiotherapist as well as further pharmaceutical adjustments or new contacts with the municipal liaison for reassessment of service needs. The type and frequency of follow-up contacts were also decided. Importantly, the plan could vary widely between participants and range from a brief period of daily contacts with the nurse and extra appointment with the doctor, to scheduled 6 monthly phone calls or visits after 1 year by a nurse (in cases where no earlier contact was deemed necessary).

The patients remained under the care of the interdisciplinary team for 24 months after recruitment. They retained access to all usual care including their primary healthcare physician and other specialist as needed, including all emergency services. All participants were given a phone number to the team nurse and if necessary other members of the team, during office hours (weekdays 8am–4.30 pm).

#### Control group (CG)

The control group received usual care provided by the Swedish healthcare system, including services provided by the municipalities.

### Outcomes

#### Primary outcome

The primary outcome was the number of days each participant spent in hospital during the 24-month follow-up, obtained from the Patient Administrative Register of Care in Region Skane [19].

#### Secondary outcomes

-All cause mortality, as registered in the Swedish cause of death register [22].

-Quality of life, as measured by the Swedish version [23] of the 2009 EuroQol Group five-dimension scale (EQ-5D-5L) [24] and a Swedish value set [25].

-Number of hospital admissions. Assessed through the Skane health care register [19].

-Physical performance as assessed using the Short Physical Performance Battery (SPPB) [26] translated into Swedish [27].

-Frailty according to the 9 level Clinical Frailty Scale (CFS), version 1.2, translated into Swedish [28]. CFS is a validated screening tool for frailty that focuses on independence, mobility, balance, and cognition.

- Activities of daily living (ADL), as measured with Katz Extended ADL Index [29] which includes 6 personal and 4 instrumental ADL measurements.

-Cognition, as measured with the Swedish version of the Montreal Cognitive Assessment (MOCA) tool [30].

#### Data collection

Data were collected through various national and regional health care registers as described above. Research nurses without any clinical relation to the participants collected additional data during home visits according to a protocol (Appendix 1). Please see specifications for each outcome measure.

### Changes to outcome

None.

### Statistical analysis

To compare the intervention and control group’s demographic data, the Chi-square test was used. It was also used to compare SPPB, ADL, and MOCA measurements in the two groups. The mean *EQ-5D-5L* values were compared using a t test. The mean number of days spent in hospital, number of hospital admissions, and frailty in the groups were compared using the Mann–Whitney U test. Mortality was analysed using Kaplan–Meier survival curves of the two groups and the hazard ratio for the intervention group was calculated using univariate Cox proportional hazards model.

The significance level was set at *p* < 0.05 for all analyses. Analysis was conducted according to the intention to treat principle, i.e., including all randomised patients regardless of actual treatment received. A per-protocol analysis was also done for the primary outcome.

## Results

### Recruitment

**Fig. 1:**
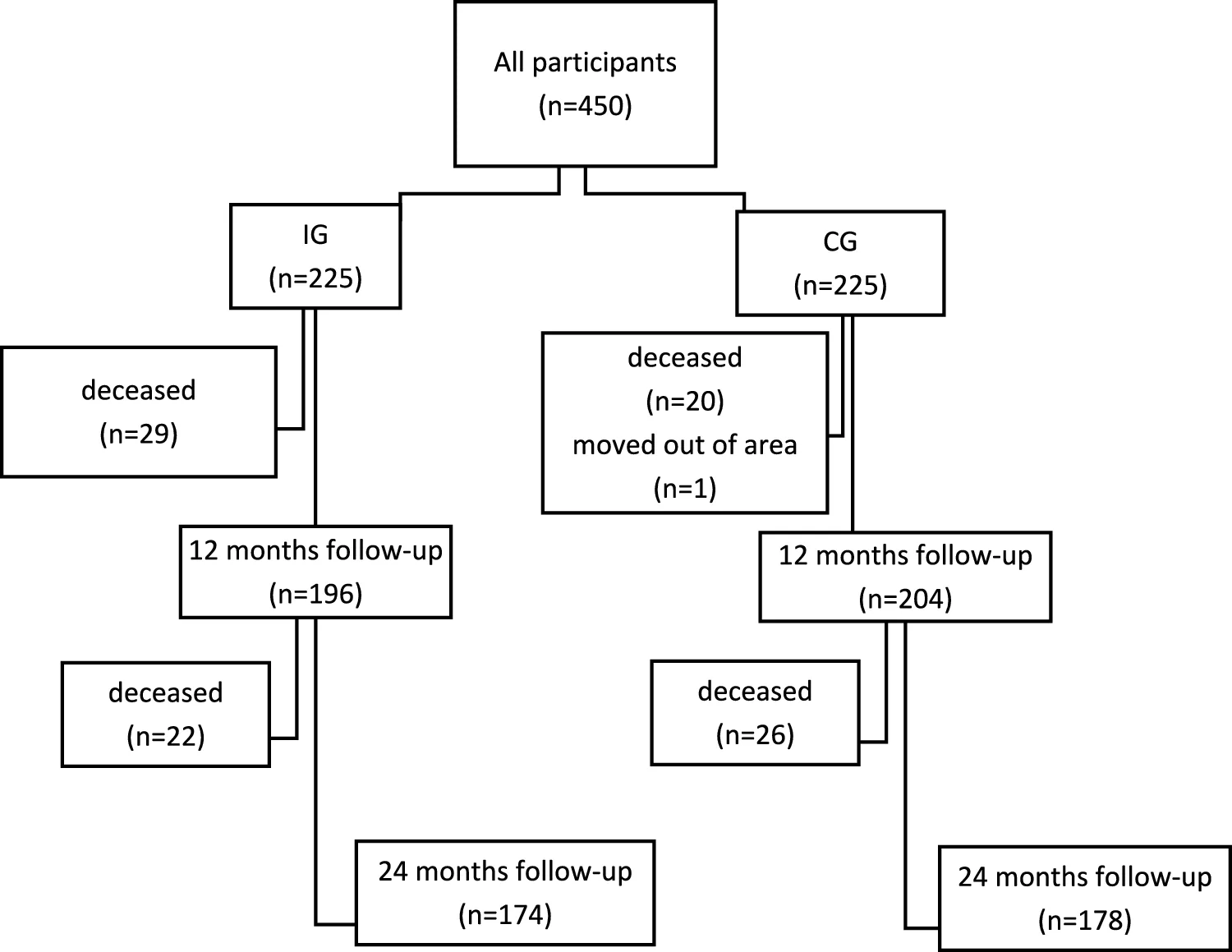
Participant flow

The recruitment took place between October 2016 and June 2018. During this time, 450 people were recruited to the study and then randomised equally to either the intervention group (*n* = 225) or the control group (*n* = 225). Follow-up occurred at 12 and 24 months after randomization. Figure 1 shows the number of participants in each group during the study. One person moved out of area during the first 12 months of the study and was therefore lost to follow-up. All other participants were followed up. Participants that during the study moved to a nursing home remained in the study.

### Baseline data

The mean age of all participants at baseline was 83 years and 245 were female (54%). More than half of the participants lived alone and 55% had not completed any secondary education. Other demographic data can be found in Table 1. As seen in Table 3, participants were, at baseline, predominantly mildly frail but had a mean MOCA score of 22. The mean SPPB score was 6 out of a maximum of 12 and the mean quality of life score (EQ-5D-5L score) was similar in the two groups; 0.75 and 0.76 in the IG and CG, respectively. The groups were of equal size, and there were no significant differences in the recorded characteristics between the interventions and control groups.

**Table 1:** Demographic data at baseline

Variable		IG *n* = 225	CG *n* = 225	*p*
Age	Mean, years [SD]	83 [5.6]	82 [5.4]	*0.58*
Height	Mean, cm [SD]	168 [11]	168 [10]	*0.42*
Weight	Mean, kg [SD]	74 [15]	74 [17]	*0.98*
Sex, *n* (%)	Male	100 (44)	105 (47)	*0.52*
Marital status, *n* (%)	Married/living with partner	115 (51)	96 (43)	
	Widow/widower	66 (29)	82 (36)	
	Divorced, not remarried	31 (14)	38 (17)	
	Never married	13 (6)	9 (4)	
				*0.18*
Accommodation, *n* (%)	Rental	84 (38)	100 (44)	
	Own apartment	71 (32)	71 (32)	
	Own house	65 (29)	52 (23)	
	Other	2 (1)	2 (1)	
				*0.42*
Living, *n* (%)	With partner/spouse	109 (49)	92 (41)	
	Alone	111 (50)	132 (59)	
	With children or other	3 (1)	1 (0)	
				*0.12*
Education, *n* (%)	Incomplete primary education	78 (35)	84 (38)	
	Complete primary education	44 (20)	40 (18)	
	Complete secondary education	19 (8)	10 (5)	
	Folk high school/vocational Education	35 (16)	47 (21)	
	University/higher education With diploma	46 (20)	38 (17)	
	Incomplete higher education or other	3 (1)	2 (1)	
				*0.32*

*CG* control group, *IG* intervention group, *SD* standard deviation

*p* value calculated using Chi-square test

### Outcomes

#### Primary outcome

At 24-month follow-up, the recorded mean cumulative number of days spent in hospital was not significantly different in the interventions and control groups. The same was true after 12 months of follow-up. The comparison can be seen in Table 2.

**Table 2:** Number of days spent in hospital during first and second year of follow-up

Follow-up	Outcome	IG *n* = 152	CG *n* = 166	*p*	*Cohen's d [95% CI]*
During month 1–12	Mean, days [SD]	8.7 [14.6]	9.6 [19.5]	0.44	−0.05 [−0.27, 0.17]
	Median, day [range]	3 [0–116]	2 [0–211]		
During month 13–24	Mean, days [SD]	7.4 [14.8]	8.0 [15.5]	0.18	−0.04 [−0.26, 0.18]
	Median, day [range]	0 [0–101]	0 [0–120]		
Total during 24 months	Mean, days [SD]	16.2 [22.3]	17.6 [25.5]	0.14	−0.06 [−0.28, 0.16]
	Median, day [range]	6 [0–116]	9 [0–211]		

During months 1–12, the intervention group showed slightly fewer days than the control group, but the effect was very small and not statistically significant. Similar negligible effects were observed during months 13–24 and for the total 24-month period.

#### Secondary outcome

**Fig. 2:**
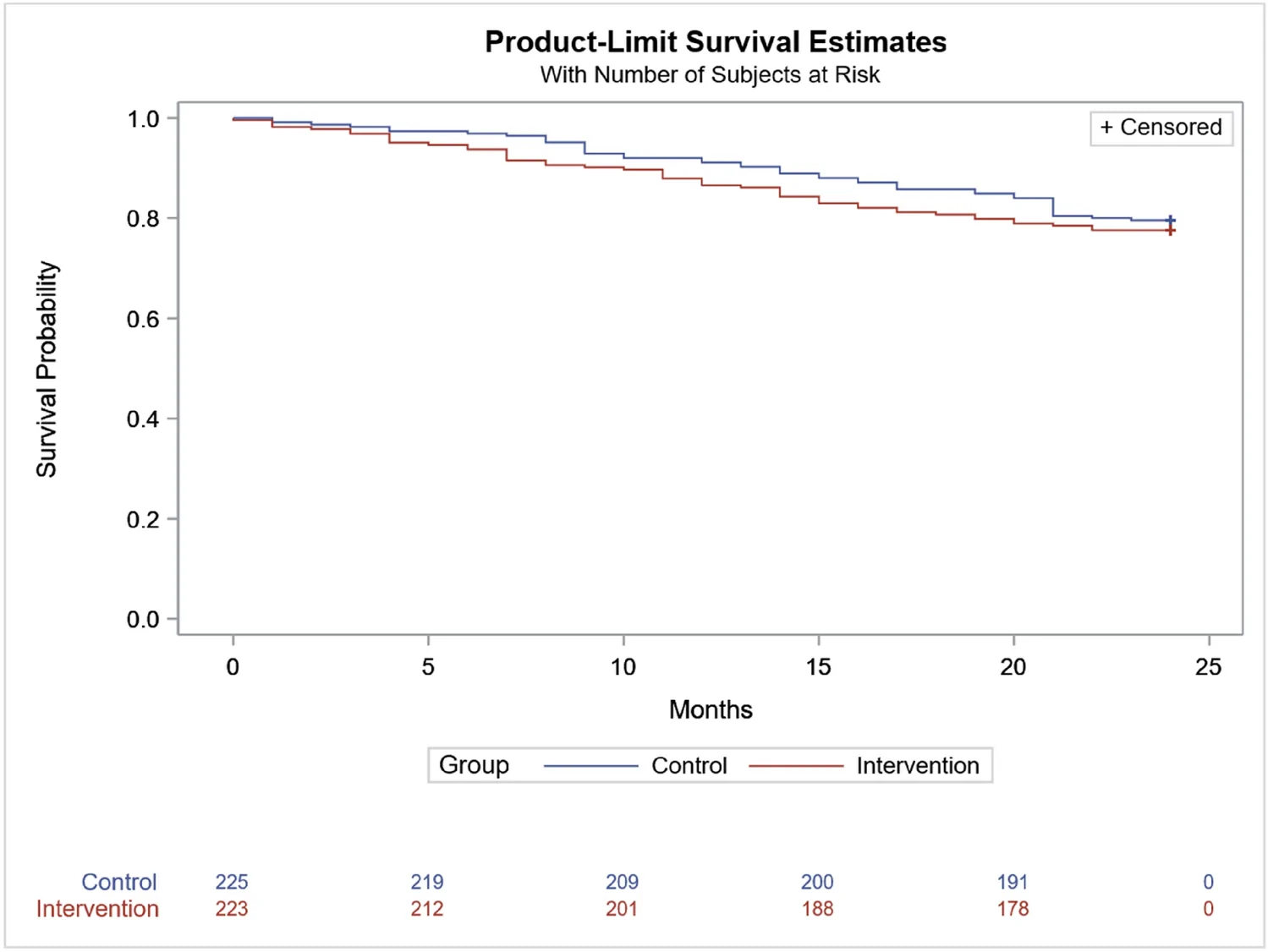
Kaplan–Meier survival plot, mortality 1–24 months post-study inclusion

There was no difference in mortality between the groups, hazard ratio 1.14 (95% CI 0.77–1.71); *p* = 0.51, Fig. 2. Quality of life, number of hospital admissions, physical performance, frailty level, and ability to perform activities of daily living or cognition also did not differ significantly between the intervention and control group at 12- or 24-month follow-up. This is also shown in effect sizes although very small effects favouring IG in later follow-ups in EQ-5DL and SPPB total score. This is presented in Table 3.

**Table 3:** Secondary outcomes presented by groups at baseline, within months 1–12 and months 13–24

		Baseline			Month 1–12			Month 13–24		
Outcome	IG	CG	*p*	IG	CG	*p*	IG	CG	*p*	
EQ-5D-5L number in group, mean value [SD]	221 0.75[0.25]	220 0.76[0.25]	*0.81*^*a*^	181 0.74[0.25]	197 0.79[0.23]	*0.13*^*a*^	160 0.74[0.29]	161 0.80[0.21]	*0.24*^*a*^	
Cohen's *d* [95% CI]		−0.04 [−0.23, 0.15]			−0.21 [−0.41, −0,01]			−0.24 [0.46, −0.02]		
No. of hospital admissions, deceased excluded mean, days [SD]	n/a	n/a		1.1[1.4]	1.4[2.2]	*0.29*^*b*^	1.1[1.7]	1.0[1.6]	0.40^b^	
Cohen's *d* [95% CI]					−0,16 [−0.36, 0.04]			0.25 [0.03, 0.47]		
SPPB total score number in group	225	225		182	200		153	155		
mean score [SD]	6 [4]	6 [4]	*0.92*^c^	6 [4]	6 [4]	*0.93*^c^	6 [4]	5 [4]	0.66^c^	
Cohen's *d* [95% CI]		0.00 [−0.18, 0.18]			0.00 [−0.20, 0.20]			0.2 [0.03, 0.47]		
CFS number in group mean score	219	219		182	199		164	169		
	4.12	4.08	*0.74*^*b*^	4.39	4.50	*0.71*^*b*^	4.95	5.15	0.34^b^	
Cohen's *d* [95% CI]		0.02 [−0.17, −0.21]			0.95 [0.26, 0.15]			−0.11 [−0.32, −0.11]		
ADL number in group mean score [SD]	222	223		178	198		160	164		
	5.3 [5]	5.5 [5]	*0.73*^*c*^	5.3 [5]	5.5 [5]	*0.55*^*c*^	6.0 [5]	6.5 [5]	0.38^c^	
Cohen's *d* [95% CI]		−0.04 [−0,23, 0.15]			−0.04 [0.24, 0.16]			−0.10 [0.32, 0.12]		
MOCA number in group mean score [SD]	221	217		179	196		147	148		
	22 [6]	22 [5]	*0.33*^*c*^	22 [6]	22 [6]	*0.77*^*c*^	22 [7]	22 [6]	*0.69*^*c*^	
Cohen's *d* [95% CI]		0.00 [−0.19, 0.19]			−0.20 [−0,20, 0.20]			0.00 [0.23, 0.23]		

*CG* control group, *IG* intervention group, *EQ-5D-5L* 5-level versions of EQ-5D (a standardised measure of health status developed by the EuroQol Group)

^a^*p* value calculated using two-sided T test

^b^*p* value calculated using Mann–Whitney U test. *SD* standard deviation. *n/a* not applicable, *SPPB* Short Physical Performance Battery

^c^*p* value calculated using Chi-Square test. *CFS* 9 level clinical frailty scale. *ADL* activities of daily living measured with Katz Extended ADL Index, *MOCA* Montreal cognitive assessment tool

#### Per protocol analysis

Of the participants randomised to the intervention group, 12 participants never took part in the intervention after baseline assessment. The per-protocol analysis excluded these 12 participants and the outcomes are presented in Table 4. This analysis did not show any significant difference between the groups in the number of days spent in hospital.

**Table 4:** Number of days spent in hospital during first and second year of follow-up (a per-protocol analysis)

Follow-up	Outcome	IG *n* = 152	CG *n* = 166	*p*	*Cohen's d [95% CI]*
During first 12 months	Mean, days [SD]	8.8 [14.9]	9.6 [19.5]	0.48	−0.05 [−0.27, 0.17]
	Median, day [range]	2 [0–116]	2 [0–211]		
During second 12 months	Mean, days [SD]	7.2 [14.7]	8.0 [15.5]	0.13	−0.05 [−0.27, 0.17]
	Median, day [range]	0 [0–101]	0 [0–120]		
Total during 24 months	Mean, days [SD]	16.1 [22.1]	17.6 [25.5]	0.14	−0.06 [−0.28, 0.16]
	Median, day [range]	6 [0–116]	9 [0–211]		

*CG* control group, *IG* intervention group, *SD* standard deviation

*P* value calculated using Mann–Whitney U test

All effect sizes were trivial (Cohen’s d ≪ 0.2), and the confidence intervals were consistent with negligible effects (Table 4). The results are consistent with the non-significant *p* values already reported.

## Discussion

In this RCT investigating the effect of CGA-based care delivered by a mobile team, no significant differences were observed between the intervention and control groups for primary or secondary outcomes after 12- and 24-month follow-up. The intervention and control groups were similar in size and baseline demographics. This suggests adequate randomisation and cannot account for the lack of significant results.

For the primary outcome, the mean number of days spent in hospital, the intervention and control groups had similar results. This can be compared to our previous RCT, AGe-FIT [17], in which the IG similarly received home CGA-based care for 24 months in addition to usual care and the CG received usual care only. AGe-FIT was conducted earlier, between 2011 and 2013, in a different part of Sweden but had the same inclusion criteria and participants had similar baseline demographics compared to the participants in GerMoT. The IG in AGe-FIT experienced significantly fewer mean number of days spent in hospital compared to the CG after 24- and 36-month follow-up [17, 18]. We speculate that the failure to replicate these results in GerMoT could stem from multiple factors.

One known difference between the interventions in GerMoT and the AGe-FIT study was the absence of a social worker in GerMoT. This might have made social planning more difficult in GerMoT; however, we did not measure this. We did not quantify this, but we speculate that there was limited geriatric experience, and less continuity of nurses, occupational therapists, and physiotherapists in GerMoT compared to Age-FIT. The functionality of multidisciplinary teams, as employed in Comprehensive Geriatric Assessment (CGA), is essential for its quality and effective implementation [31]. The high staff turnover in the newly established geriatric ward—from which GerMoT operated—alongside a possible lack of geriatric experience among newly recruited personnel, we hypothesis, could have played a role in the quality of the intervention. In addition, team building was not a central component of this intervention as no nurse, physiotherapist, occupational therapist, or physician other than the principal investigator remained continuously involved in the intervention. Although a project leader was appointed at the outset, there was no consistent daily leadership, and the appointed individual left early in the project. We speculate that these factors might have led to a limited understanding of the importance of ongoing team training [32]. This is in contrast to our previous randomized controlled trial AGe-FIT[17], where structured team building activities—including excursions, social gatherings, and collocating team members in the same workspace—took place. Team training has also been shown to improve adherence to clinical guidelines [33].

A possible lesson from this study is the likely importance of continuous team building and development, as emphasized in prior literature [31–33]. Future implementations of CGA should prioritize structured team training to ensure both effective collaboration and fidelity to intervention protocols.

The participants had a mean baseline CFS score of around 4 (IG 4.12, CG 4.08) indicating that many participants were not frail. Many trials on the effects of CGA [7] predate widespread use of tools like the CFS. However, they consistently highlight that CGA effects are more pronounced in frailer subgroups. In addition, terminally ill individuals do not see much benefit. The lack of effect in this trial might also be explained in part by many participants being non-frail. The inclusion criteria were chosen to be the same as in our former study AGe-FIT [17] and were based on information available in the electronic Patient Administrative Register of Care in Region Skane [19]. This database had no information on frailty; therefore, recruitment based on frailty would have required a relatively resource intensive prior assessment of frailty. In the future, electronical records will probably contain measures of frailty, making it possible to include only frail individuals.

The lack of differences in both primary and secondary outcomes between the intervention and control groups could also have been caused by the control group receiving elements of CGA-based care. Many recent studies with “usual care” as the control do not show as much effect of CGA as earlier trials. This may reflect integration of CGA elements into standard guidelines and usual care over time [34]. This might also have been the case in this trial.

Future studies on outpatient CGA compared to usual care, as discussed above, might be less likely to show significant results for the outcomes we studied as modern usual care has incorporated elements of CGA. Future research should shift from a focus on health outcomes and healthcare consumption to studying CGA implementation, co-operation between health and social care providers, and resource allocation. [35, 36].

This study has several possible limitations. First, we can see that there was a large variation in the number of days spent in hospital for participants within the intervention and control groups. This heterogenicity in outcomes within each group could reflect that there are multiple factors that affect hospital admissions and length of stay in hospital. Therefore, one hypothesis is that our study might have had insufficient power to register an effect of our intervention.

Secondly, the GerMoT trial studied a specific group in one geographic area. Although we suspect that many generalisations can be made about people fulfilling these criteria but living elsewhere, there might be significant differences based on geographic locations.

Third, the intervention group received CGA-based outpatient care according to the general principles of CGA; however, this might have been limited by continuity and competence available at the trial site. We did not measure the fidelity of the intervention so can only speculate that it has impacted the outcomes.

Finally, this study evaluated the effect of the intervention on the number of days spent in hospital. While hospitalization is sometimes necessary, reducing hospital days is clinically beneficial only when it targets avoidable hospitalisations. A more relevant outcome would have been the number of avoidable hospitalisations in the intervention and control groups. The primary outcome in this study, although easier to measure, serves only as a proxy to this. We assumed that reducing hospital days corresponds to a reduction in avoidable hospitalisation; however, this assumption might not always hold true. In fact, preventing necessary hospitalisation might have been detrimental to patients. The outcomes we recorded were not worse in the IG than the CG; however, we can only speculate about potential other harms which we have not measured.

A strength of GerMoT is its real-life setting. Anyone designing a CGA-based mobile team interventions might encounter staffing, competence, continuity, and resource shortages. This is reflected in the difficulties faced during GerMoT. GerMoT featured a robust recruitment process [20], with a high consent rate, broad inclusion criteria, no upper age limits, and included participants with cognitive disorders. Attempts were made to contact all people meeting the inclusion criteria in the area. These factors ensured a representative sample.

## Conclusions

This trial found that CGA-based care delivered in patient’s homes by a mobile team, alongside usual care, did not significantly affect the number of days spent in hospital, mortality, quality of life, number of hospital admissions, physical performance, frailty, activities of daily living (ADL), and cognition after 2 years of follow-up. We hypothesis that the lack of continuity of staff and lack of team building might have affected the results. Besides, many elements of CGA have probably already been incorporated into usual primary care. Future research should focus on how to further implement CGA into usual care including how to maintain continuity of the care team.

## Protocol

The study protocol has been published [21].

## Data Availability

This research is part of a PhD thesis at Lund University and is in deposit at the University’s repository. The datasets generated and analysed during this study are stored by Anne Ekdahl and available from the corresponding author on reasonable request.
